# Combined Therapy with Azathioprine, Prednisone, and Enalapril in Children with IgAN and IgAVN

**DOI:** 10.3390/jcm13237316

**Published:** 2024-12-02

**Authors:** Małgorzata Mizerska-Wasiak, Miłosz Starczyński, Wojciech Wasiak, Jadwiga Małdyk, Emilia Płatos, Małgorzata Pańczyk-Tomaszewska

**Affiliations:** 1 Department of Pediatrics and Nephrology, Medical University of Warsaw, 02-091 Warsaw, Poland; mpanczyk1@wum.edu.pl; 2 Student’s Scientific Group, Department of Pediatrics and Nephrology, Medical University of Warsaw, 02-091 Warsaw, Poland; milosz.starczynski@wum.edu.pl (M.S.); wojtek.wasiak@gmail.com (W.W.); emilia.platos@wum.edu.pl (E.P.); 3 Department of Pathology, Medical University of Warsaw, 02-091 Warsaw, Poland

**Keywords:** IgAN, IgAVN, combined treatment, azathioprine, prednisone, enalapril

## Abstract

**Background**: The aim of this study was to evaluate the efficacy of 1-year treatment in children with IgAN and IgAVN using azathioprine, prednisone, and enalapril (AZA+PRED+E) combined with a control kidney biopsy. **Methods**: This study consists of 68 children diagnosed via kidney biopsy with Oxford classification. The study group included 36 children (15 IgAN, 21 IgAVN) treated with AZA+PRED+E (according to the protocol with a control kidney biopsy); and the control group included 32 children (21 IgAN, 11 IgAVN) who were treated with enalapril alone during one year after kidney biopsy. **Results**: After 1 year of combined therapy, a significant reduction in both proteinuria (proteinuria = 0 in 35 patients from the study group) and hematuria in the study group was found. It was confirmed that the Δ proteinuria between the start and end of treatment in IgAN and IgAVN patients from the study group was significantly higher than the Δ proteinuria between the start and end of treatment in the control IgAN and IgAVN group treated with enalapril (30.7 ± 43.6 vs. 8.7 ± 8.7; *p* = 0.015; 68.2 ± 58.3 vs. 19.3 ± 20.3; *p* = 0.008 respectively). In the Oxford classification a high frequency of improvement in E and T in the study group after treatment was observed. **Conclusions**: Patients with higher proteinuria and a higher MESTC score require consideration of the strategy of immunosuppressive treatment so that therapy with AZA+PRED+E may be used as a personal treatment plan for children with these diseases.

## 1. Introduction

IgA nephropathy (IgAN) is the most frequently diagnosed type of primary glomerulonephritis worldwide. The diagnosis, sometimes based only on kidney biopsy results, is characterized by the presence of isolated IgA deposits under immunofluorescence or by the predominance of IgA deposits over others. The histopathological picture of IgA nephropathy was also identified in IgA vasculitis nephritis, IgAVN. Thus, some define them both as IgA nephropathies [1,2], although they should be considered as two different disease entities [3]. According to Kiryluk et al.’s results, the frequency of the alleles responsible for IgAN varies depending on the geographical location. Additionally, the course of the disease prognosis differs between Asian and European cohorts [4,5].

Currently, the progression of lesions in kidney biopsy samples defined by the Oxford classification of IgA nephropathy, consisting of parameters presenting in histopathology, has the most unfavorable prognostic significance [6]. This classification might also be helpful in determining the indications for treatment implementation in IgA nephropathy: immunosuppression, glucocorticoid therapy, or renoprotection, as well as its evaluation [7].

The WHO classification with Grades I–V is among the older and less often-used scales [8]. This scale does not include prognostic factors, although interstitial lesions—present in Grades III to V—are considered to indicate a poor prognosis.

A standardized treatment of nephropathy in children does not exist; therefore, various methods have been applied and described thus far. The early administration of angiotensin-converting enzyme inhibitors (ACE inhibitors) is recommended for all patients due to their renoprotective effect. They slowed down the development of kidney insufficiency and reduce the intensity of proteinuria [9]. Also, Omega 3 unsaturated fatty acids in fish oil have beneficial effects, although the current KDIGO guidelines allow but do not recommend its implementation [9].

According to the Cochrane 2020 metanalysis, the influence of any other immunosuppressive scheme on the clinical outcomes of IgA nephropathy (local glucocorticosteroids, antiproliferative, mycophenolate mofetil, calcineurin inhibitors, mizoribine, leflunomide, and mTOR inhibitors with or without steroids) remains uncertain. The data were irrelevant either due to a small number of studies or clinically insignificant results [10]. 

Recommendations for the treatment of pediatric glomerulonephritis are currently in preparation, although the 2021 KDIGO guidelines provide valuable guidance for the treatment of glomerulonephritis primarily in adults. Some believe that immunosuppressive treatment for children with IgAN should not be considered in the same way as in adult IgAN, because the KDIGO guidelines were created for patients over 18 years [11].

Only a few papers have shown the effects of pediatric treatment with a control renal biopsy. The aim of the study was to evaluate the efficacy of 1-year treatment in children with IgAN and IgAVN using azathioprine, prednisone, and enalapril combined with a control renal biopsy as a possible personal plan of treatment of these diseases.

## 2. Materials and Methods

From the group of 123 children with IgAN and IgAVN, only individuals treated with azathioprine combined with prednisone and ACE inhibitors, as well as a control kidney biopsy after 12 months of therapy, and those treated with enalapril alone for 12 months were included. The diagnosis was based on kidney biopsy findings and the patients remained under the control of doctors from the Department of Paediatrics and Nephrology at the University Hospital between 1999 and 2019.

The indication for initiation of the treatment was proteinuria and Grade II–IV in the WHO scale of lesion progression in kidney biopsy samples at the onset of disease: non-nephrotic proteinuria and WHO ≥ III, or nephrotic proteinuria and WHO ≥ II. Azathioprine was administered at a dose of 2 mg/kg/day for a period of 12 months combined with prednisone at a dose of maximum 60 mg/day which was gradually reduced during a period of 15 months. Additionally, enalapril was administered in the maximum tolerated dose in the study group.

Matching criteria were as follows: diagnosis of IgAVN—patient should meet inclusion criteria for treatment with azathioprine, prednisone, and enalapril, same length of treatment (azathioprine—12 months; prednisone—15 months; enalapril—15 months), and control kidney biopsy after treatment. Ultimately, 15 children with IgAN and 21 patients with IgAVN meeting the above criteria were included in the study group and 21 children with IgAN and 11 children with IgAVN met the criteria of the control group.

This study was retrospective. Patients were qualified for a specific type of treatment according to the approved protocol. Only patients who completed a 1-year treatment and had a follow-up renal biopsy were eligible for analysis. Individuals without a control renal biopsy were excluded from the study. All patients consented to a first and follow-up kidney biopsy.

The size of the study and control groups was determined by the number of patients who met the inclusion criteria, covering all patients treated during the study period as shown at the beginning and the end of treatment. Protein and erythrocytes in the urine, and creatinine, IgA, and C3 in the blood, were measured, and the GFR was calculated. Proteinuria in the urine collected over 24 h was measured using the Exton turbidimetric method and expressed in mg/kg/day. Nephrotic proteinuria (NP) was detected above the level of 50 mg/kg/day of protein in a urine sample. Non-nephrotic proteinuria (NNP) was detected up to the level of 50 mg/kg/day. Hematuria was detected when more than five erythrocytes were visible. Gross hematuria was considered in the case of a change in the color of the urine. In total, 200 erythrocytes being visible was considered to classify hematuria and 250 for gross hematuria. Creatinine in serum was measured with the Vitros 250 in dry chemistry tests and expressed in mg/dl. GFR was calculated based on the Schwartz formula in mL/min/1.73 m^2^ [12]. 

A kidney biopsy was performed on every patient before and after azathioprine implementation. The histopathological specimens were evaluated in optic microscopy with the use of the WHO scale and retrospectively in the Oxford classification [6] with immunofluorescence. IgA nephropathy was identified by the presence of IgA deposits exclusively or their predominance over other deposits.

WHO classification Grades I–IV include the following: Grade I—minimal changes; Grade II—mesangial proliferation, 50% of glomeruli, rare small crescents, no changes in the interstitium; Grade III—diffuse mesangial proliferation, small crescents, foci of vascular loop adhesion, limited edema, and infiltrates in the interstitium; Grade IV—proliferation and hyalinization involving all glomeruli, increased number of mesangial cells, crescents and focal capsular vascular loop adhesion in < 50% of glomeruli, infiltrates in the interstitium, and tubular atrophy; Grade V—segmental or complete glomerular hyalinization, crescents in > 50% of glomeruli, infiltrates in the interstitium, and tubular atrophy. 

The Oxford classification evaluates the following: 

Mesangial hypercellularity—< or >50% glomeruli = M0 or M1;

Endocapillary hypercellularity—present or absent = E0 or E1;

Segmental sclerosis/adhesion—present or absent = S0 or S1;

Tubular atrophy/interstitial fibrosis—0–25%, 26-50%, or 50% = T0, T1, or T2, respectively;

Crescents—absent, 0–25%, >25% = C0, C1, or C2, respectively.

The influence of the implemented treatment on changes in urinalysis, GFR, and kidney tissue samples was evaluated. The side effects of the therapy were evaluated in the following tests: complete blood count, GPT, GOT, and fasting glucose. In addition, arterial blood pressure, height, and weight were regularly measured, and eye examinations were performed.

### Statistics

Parametric tests were used depending on the distribution of the variable. The distributions of quantitative variables were verified with the Shapiro–Wilk test.

For variables with normal distributions, parametric tests were used: Student’s *t*-test for independent samples and Student’s *t*-test for dependent samples (to compare a given variable: first biopsy with the second biopsy). 

For variables with a non-normal distribution, the Mann–Whitney U test (independent groups) and Wilcoxon matched pairs test (dependent groups) were used.

For the remaining qualitative variables, ordinal or dichotomous (ordinal: e.g., MESTC sum, IgA total, C3 integer; dichotomous: M, E, S, T, C) non-parametric tests were used: Mann–Whitney and a sign test. The test for probability was used to check the differences between the frequency of occurrence of a given feature between HSN and IgAN.

GFR >= 90 mL/min/1.73 m^2^ was the boundary for the improvement of GFR. GFR < 90 mL/min/1.73 m^2^ was the boundary for the deterioration of GFR.

Patients with IgAN constituted the baseline for individuals with IgAVN, meeting the same inclusion criteria in the study. All patients had a complete data set.

## 3. Results

This study comprises 68 children diagnosed via kidney biopsy and 36 (15 IgAN and 21 IgAVN) who were treated with azathioprine, prednisone, and enalapril (according to the protocol with a control kidney biopsy). The control group counted 32 children (21 IgAN and 11 IgAVN) who were treated with enalapril only during one year after kidney biopsy. The clinical data of the study group and control group before the treatment are presented in Table 1.

At the onset of disease, proteinuria was significantly higher in children from the study group than in the control group, creatinine, GFR, IgA, and C3, without significant differences. 

The results of kidney biopsy at the onset of disease are presented in Table 2.

In the study group, a significantly higher MESTC score than in the control group (*p* = 0.003) was observed significantly more often for E1, T1-2, and C1-2 (*p* = 0.025, *p* = 0.002, and *p* = 0.05, respectively).

In the first biopsy, the MEST-C score was not significantly different between the IgAN and IgAVN study groups.

The data at the onset and the end of treatment for the study and control groups are presented in Table 3.

In IgAN and also in IgAVN, significantly higher proteinuria at the onset of disease was observed in the study group than in the control group (*p* = 0.04 and *p* = 0.009, respectively). 

After 1 year of combined therapy, we found a significant reduction in both proteinuria (proteinuria = 0 in 35 patients from the study group) and hematuria in the IgAN and IgAVN study groups. GFR and creatinine were normal in all patients of both groups. 

In comparison to the control group, a significantly lower hematuria in IgAVN and significantly higher GFR in IgAN at the end of the treatment (*p* = 0.05 and *p* = 0.009, respectively) was noted.

It was confirmed that the Δ proteinuria between the start and end of treatment in IgAN patients treated with azathioprine, prednisone, and enalapril was significantly higher than the Δ proteinuria between the start and end of treatment in the control IgAN group treated with enalapril alone (30.7 ± 43.6 vs. 8.7 ± 8.7; *p* = 0.015). Also, the Δ proteinuria between the start and end of treatment in IgAVN patients treated with azathioprine, prednisone, and enalapril was significantly higher than the Δ proteinuria between the start and end of treatment in the control IgAVN group treated with enalapril alone (68.2 ± 58.3 vs. 19.3 ± 20.3; *p* = 0.008).

Logistic regression was performed, which showed that the parameters significantly differentiating the treatment groups, IgAN and IgAVN, from the controls are Δ proteinuria and Δ IgA. 

The effect of age on the value of Δ IgA was also analyzed. The result of the analysis of covariance indicated that the age of onset has no effect on the differences in Δ IgA between the treatment groups vs. the control groups.

**Figure 1:**
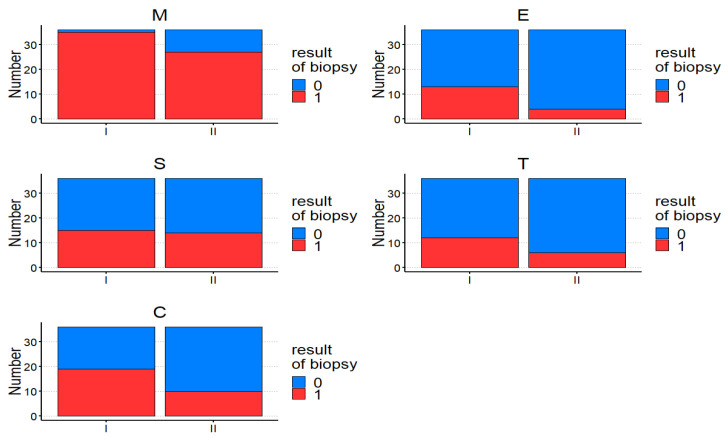
The comparison between diagnostic (I) and control (II) kidney biopsy according to the Oxford classification.

**Figure 2:**
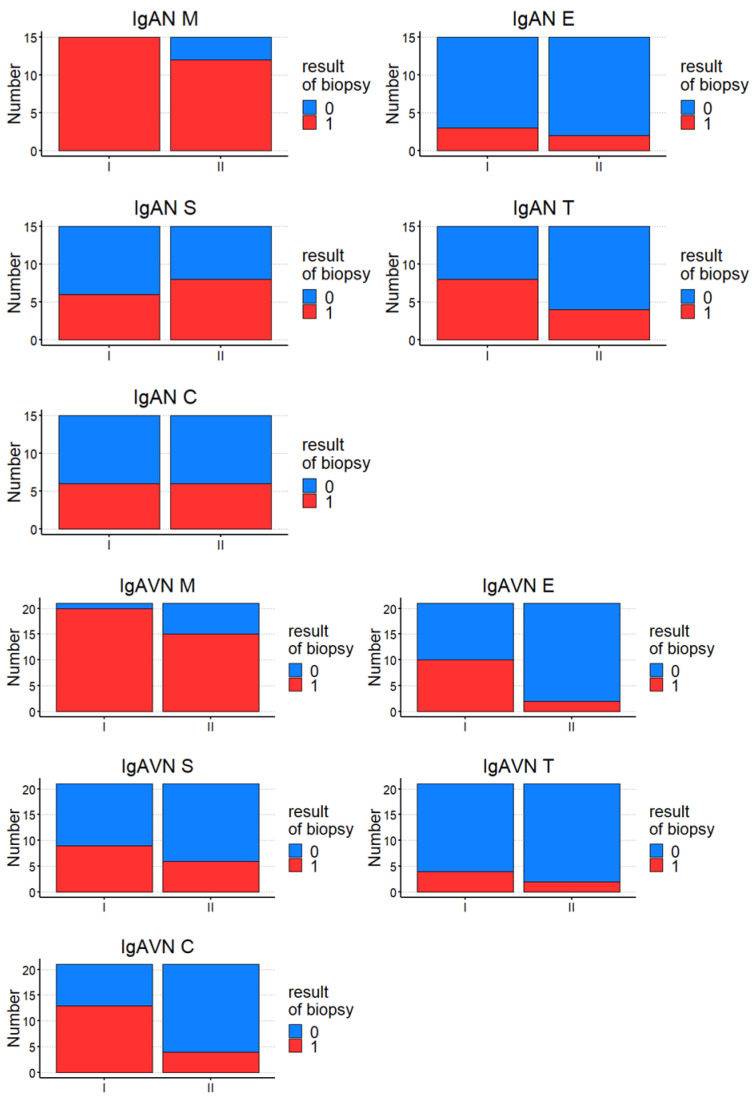
The comparison between diagnostic (I) and control (II) kidney biopsy according to the Oxford classification in IgAN and IgAVN separately.

The comparison between diagnostic and control kidney biopsy according to the Oxford classification in both groups is presented in Figure 1 and separately in Figure 2.

In the Oxford classification, significant improvement in M, E, and C between the first and second biopsy in the IgAN and IgAVN groups was noted. In IgAN, improvement but not significant improvement in M, E, and T between the first and second biopsy was noted. In IgAVN, a significant improvement in E and C after the treatment was observed. In the IgAN and IgAVN groups, the frequency of improvement was calculated, defined as Frequency of improvement (n) = Improvement or 0 – Worsening and presented in Table 4.

A high frequency of improvement in E and T in both groups and C in IgAVN after treatment was observed, without significant differences between IgAN and IgAVN. The analysis of the intensity of the depositions of immunoglobulins and complement components in the first and second kidney biopsies also showed a significant reduction in the intensity of the deposits of IgA, IgG, IgM, and C3 in the control kidney biopsy in both groups. In IgAN, a significant reduction in the intensity of deposits of IgA and C3 after treatment, and in IgAVN of deposits of IgA, was noted.

## 4. Discussion

The current KDIGO recommendations for IgAN are mainly for adult patients [9]. According to the Coppo R report, in Europe, children with IgAN have a favorable prognosis in a short time, which may also result from the frequently used cyclosporin A or immunosuppressive therapy, especially in the case of acute and active histopathological lesions [13], which in Valiga’s study was used in the treatment of more than half of the patients [14].

There is some doubt that IgAN is the same disease in children and adults. If it is, early intervention may be an appropriate way to prevent disease progression in a long-term follow-up. However, if these are different diseases, treatment in children and adults must be implemented separately [15]. The treatment of IgAN with immunosuppressants differs between adults and children, and the use of immunosuppressants is more common in children, especially glucocorticoids. However, there is a lack of randomized control trials and expert indications for the pediatric group of patients [9]. Similarly, there is a shortage of controlled trials with the analysis of the treatment implemented in IgAVN. 

Although the presented study is not a randomized control trial, a control kidney biopsy was performed at the end of the treatment, which was the only direct and objective way to evaluate its effectiveness and hence one of the few ways to tackle this problem. This study included a group of 36 patients with IgAN and IgAVN whose treatment efficacy was analyzed on the basis of clinical symptoms and kidney biopsy results after treatment implementation with the combined therapy of azathioprine, prednisone, and enalapril, and 32 children treated with enalapril. Even though a few studies containing a control kidney biopsy after immunosuppressive treatment of these diseases have been published, the follow-up kidney biopsy did not cover the entire study group. Immunosuppressive treatment was recommended among Japanese children with IgAN by Yoshikawa et al., who compared a two-year treatment with azathioprine, prednisone, Warfarin, and Dipyridamole with a two-year treatment with Warfarin and Dipyridamole in a group of 74 patients, including 40 individuals receiving azathioprine [16]. The authors of the 10-year follow-up came to similar conclusions. They proved that a two-year combination therapy not only alleviated the acute phase of nephritis but also improved the long-term outcomes of severe childhood IgA nephropathy. They believed renal function to be reversible at an eGFR of 89 to 60 mL/min per 1.73 m2 and to be irreversible at an eGFR of 60 mL/min per 1.73 m2 in the group of pediatric patients with IgA nephropathy [17]. 

Also, in Foster’s studies of a group with IgAVN, including a 1-year treatment of 20 patients with azathioprine, only 13 of them underwent a control renal biopsy. This study confirmed that early treatment with this combination therapy prevented chronic changes in kidney biopsy [18], which was also confirmed by Shin’s study on a group of 20 Korean children treated with azathioprine combined with steroids in 10 patients, or steroids alone in 10 patients, with a control kidney biopsy in 10 patients treated with azathioprine. These works showed that in the group of patients treated with azathioprine, six of them achieved clinical remission and four achieved reduced proteinuria, whereas in the group treated only with steroids, four of them achieved clinical remission, two achieved reduced proteinuria, and four showed no improvement or renal failure in long-term follow-up. The combination treatment of azathioprine and steroids might be beneficial in ameliorating histopathological features and improving the clinical course of severe HSN [19]. This study proved the potential for both clinical remission, i.e., the disappearance of proteinuria, which was found in 100% of children with IgAVN, and histopathological improvement in this group, which was 90.5% for E, 85.7% for T, and 81% for C. In the IgAN group, proteinuria disappeared in 93% of the patients and reduced in the case of one patient, and the rates of histological improvement were the following: 86,7% for E, 73,3% for T, and 53% for C. This improvement was observed despite the higher MESTC score in the study group.

The Δ proteinuria between the start and end of treatment was a very important observation. It was also confirmed that in IgAN and IgAVN patients treated with azathioprine, prednisone, and enalapril, the Δ proteinuria was significantly higher than between the start and end of treatment in the control IgAN and IgAVN groups treated with enalapril alone. This might prove the power of this immunosuppressive therapy.

This research indicates the need for a control kidney biopsy which might be helpful to distinguish active from chronic lesions and hence, according to the Oxford classification, also to indicate the proper treatment. The diagnosis of complete remission might affect treatment as well as the patient’s outcome. Therefore, it should be recommended that repeat biopsy be included in clinical practice and future clinical trials [20].

As in Shin’s and Foster’s research studies, a reduction in IgA deposits in the control kidney biopsy in groups with IgAN and IgAVN was also noticed in this study, which was associated with a reduction in proteinuria. Due to the different courses of these diseases described in the Asian and European populations, conducting the study in a European group of children is undoubtedly the value of this study. Cambier et al. suggested administering steroids at an early stage in IgAN with a global approach including active lesions, i.e., the M1 stage at least [20]. Some isolated side effects seen in Yoshikava’s study included leukopenia, mild glaucoma, mild cataract, depression, peptic ulcer, and mild elevation of transaminase concentration. Azathioprine was discontinued in this case until the side effects subsided. They observed a relevant increase in the mean obesity score in both study groups [16]. The side effects of the 1-year treatment in the studied group were as follows: weight gain, which was observed in 22 (61%) patients; leukopenia in 1 (3%), which subsided after the end of the treatment; and slow growth in 28 (78%). Significant weight gain and slow weight gain after treatment were found in the group of children with IgAVN (SDS weight -0.1 increased to 0.29 (*p* = 0.019); SDS height −0.11 to −0.33 (*p* = 0.048)), whereas in IgAN, these changes were not statistically significant (SDS weight 0.29 to 0.55 (*p* = 0.215); SDS height 0.63 to 0.2 (*p* = 0.061)). Particular attention was paid to the side effects of these therapies. According to the Italian authors Sarcina et al., a significantly higher number of effects occurred after combined therapy of cyclosporin A with azathioprine rather than cyclosporin A alone, but the study was conducted in adults [21]. Although in the studied group leukopenia occurred with a similar frequency as in the Italian research, an increased number of infections, diabetes, hepatotoxicity, or arterial hypertension (normal RR was probably related to ACE inhibitor administration) in the study group was not observed. Perhaps this therapy is much better tolerated in children than in adult patients.

The limitations of this study were its retrospective form, study group size, and lack of a control kidney biopsy in the control group. The implementation of kidney biopsy in pediatric patients of European origin after successful treatment was undoubtedly a huge advantage of this work, exceeding its limitations.

## 5. Conclusions

One year of therapy with azathioprine, prednisone, and enalapril led to a clinical and histological improvement in children with IgAN and IgAVN similar to that of the control group, despite the worse prognosis than in the control group. Therefore, this therapy should be considered as an effective treatment for these diseases. 

The choice of treatment of IgAN and IgAVN in children should be dictated by clinical symptoms and the results of a kidney biopsy. Patients with higher proteinuria and a higher MESTC score require consideration of the strategy of immunosuppressive treatment so that therapy with azathioprine, prednisone and enalapril might be used as a personal treatment plan for children with these diseases.

## Figures and Tables

**Table 1:** The clinical data of the study group and control group before the treatment.

	IgAN+ IgAVN (N = 36) START	Control IgAN + IgAVN (N = 32) START	*p*
Proteinuria mg/kg/day	16.5 (13–30)	13.5 (0–115)	0.0001
Hematuria (erythrocytes/HPF)	70 (30–250)	75 (5–250)	0.7
Creatinine (mg/dL)	0.6 (0.5–0.7)	0.6 (0.3–1.2)	0.8
GFR (mL/min/1.73 m^2^)	102.49 ± 30.1	108.37 ± 27.1	0.27
IgA (mg/dL)	311.33 ± 154.4	264.21 ± 136.42	0.29
C3 (mg/dL)	114.2 ± 26.81	110.74 ± 29.88	0.20

**Table 2:** The results of kidney biopsy at the onset of disease in the IgAN, IgAVN, and control groups.

	Study IgAN + IgAVN	Control IgAN + IgAVN	*p*
Time to biopsy (years)	1.03 ± 1.03	0.11 ± 0.11	0.0002
MESTC sore	3.0 (1–5)	1.0 (0–3)	0.003
M1 (%)	97	90	0.25
E1 (%)	36	12	0.025
S1 (%)	42	16	0.187
T1-2 (%)	33	3	0.002
C1-2 (%)	64	38	0.05

**Table 3:** The data at the onset and the end of the treatment for the study and control groups.

	IgAN (N = 15)	Control	*p*	IgAVN (N = 21)	Control	*p*
**START treatment**						
Proteinuria mg/kg/day	16.5 (0–177)	12.7 (0–23)	**0.04**	43 (5.79–190)	13.8 (0–115)	**0.009**
Hematuria (erythrocytes/HPF)	70 (30–250)	200 (5–250)	0.73	40 (30–80)	35 (5–250)	0.45
Creatinine (mg/dL)	0.6 (0.5–0.7)	0.7 (0.4–1.2)	0.92	0.5 (0.42–0.6)	0.5 (0.3–0.7)	0.31
GFR (mL/min/1.73 m^2^)	102.49 ± 30.10	102.07 ± 25.58	0.96	127.15 ± 35.31	119 ± 27.14	0.55
IgA (mg/dL)	311.33 ± 154.4	311.10 ± 124.90	0.99	295.44 ± 158.28	178.95 ± 117.45	**0.04**
C3 (mg/dL)	114.20 ± 26.81	107.65 ± 24.90	0.54	123.32 ± 24.15	116.36 ± 19.82	0.42
**STOP treatment**						
Proteinuria mg/kg/day	0 (0 -0)	0 (0–25)	0.48	0 (0–6)	0 (0–0)	0.856
Hematuria (erythrocytes/HPF)	0 (0–6)	10.5 (0–200)	**0.05**	1 (0–5)	0 (0–10)	0.15
Creatinine (mg/dL)	0.7 (0.6–0.8)	0.7 (0.4–1.0)	0.22	0.5 (0.4–0.6)	0.5 (0.4–0.8)	0.85
GFR (mL/min/1.73 m^2^)	122.48 ± 28.49	100.91 ± 18.66	**0.009**	122.88 ± 25.99	112.64 ± 19.39	0.28
IgA (mg/dL)	223.49 ± 143.47	321.58 ± 104.96	0.03	207.71 ± 86.65	159.36 ± 133.10	0.24
C3 (mg/dL)	102.26 ± 26.54	103.08 ± 24.39	0.92	93.17 ± 23.28	94.73 ± 13.24	0.84

**Table 4:** Frequency of improvement after treatment in children with IgAN and IgAVN.

	M	E	S	T	C
**IgAN**	1 (0.067)	10 (0.667)	3 (0.2)	8 (0.533)	7 (0.467)
**IgAVN**	6 (0.286)	17 (0.81)	12 (0.571)	18 (0.857)	17 (0.81)
*p*	*p* = 0.1	*p* = 0.32	*p* = 0.026	*p* = 0.0324	*p* = 0.031

## Data Availability

No new data were created or analyzed in this study. Data sharing is not applicable to this article.

## References

[1] Coppo R., D’Amico G. (2005). Factors predicting progression of IgA nephropaties. J. Nephrol..

[2] Wyatt R.J., Julian B.A. (2013). IgA nephropathy. N. Engl. J. Med..

[3] Pillebout E., Sunderkötter C. (2021). IgA vasculitis. Semin. Immunopathol..

[4] Gharavi A.G., Kiryluk K., Choi M., Li Y., Hou P., Xie J., Sanna-Cherchi S., Men C.J., Julian B.A., Wyatt R.J. (2011). Genome-wide association study identifies susceptibility loci for IgA nephropathy. Nat. Genet..

[5] Zhang H., Barratt J. (2021). Is IgA nephropathy the same disease in different parts of the world?. Semin. Immunopathol..

[6] Roberts I.S., Cook H.T., Troyanov S., Alpers C.E., Amore A., Barratt J., Berthoux F., Bonsib S., Bruijn J.A., Working Group of the International IgA Nephropathy Network and the Renal Pathology Society (2009). The Oxford classification of IgA nephropathy: Pathology definitions, correlations and reproducibility. Kidney Int..

[7] Yamamoto R., Imai E. (2009). A novel classification for IgA nephropathy. Kidney Int..

[8] Davison A.M., Cameron J.S., Grünfeld J.P., Ponticelli C., Winearls C.G., Van Ypersele C. (1998). IgA nephropaties. Oxford Textbook of Clinical Nephrology.

[9] Rovin B.H., Adler S.G., Barratt J., Bridoux F., Burdge K.A., Chan T.M., Cook H.T., Fervenza F.C., Gibson K.L., Glassock R.J. (2021). Kidney Disease: Improving Global Outcomes (KDIGO) Glomerular Diseases Work Group. KDIGO 2021 Clinical Practice Guideline for the Management of Glomerular Diseases. Kidney Int..

[10] Natale P., Palmer S.C., Ruospo M., Saglimbene V.M., Craig J.C., Vecchio M., Samuels J.A., Molony D.A., Schena F.P., Strippoli G.F. (2020). Immunosuppressive agents for treating IgA nephropathy. Cochrane Database Syst. Rev..

[11] Cambier A., Boyer O., Deschenes G., Gleeson J., Couderc A., Hogan J., Robert T. (2020). Steroid therapy in children with IgA nephropathy. Pediatr. Nephrol..

[12] Schwartz G.J., Mun A., Schneider M.F., Mak R.H., Kaskel F., Warady B.A., Furth S.L. (2009). New equations to estimate GFR in children with CKD. J. Am. Soc. Nephrol..

[13] Coppo R. (2019). Pediatric IgA Nephropathy in Europe. Kidney Dis..

[14] Coppo R., Lofaro D., Camilla R.R., Bellur S., Cattran D., Cook H.T., Roberts I.S.D., Peruzzi L., Amore A., Emma F. (2017). Risk factors for progression in children and young adults with IgA nephropathy: An analysis of 261 cases from the VALIGA European cohort. Pediatr. Nephrol..

[15] Coppo R. (2021). Treatment of IgA nephropathy in children: A land without KDIGO guidance. Pediatr. Nephrol..

[16] Yoshikawa N., Ito H., Sakai T., Takekoshi Y., Honda M., Awazu M., Ito K., Iitaka K., Koitabashi Y., Yamaoka K. (1999). A controlled trial of combined therapy for newly diagnosed severe childhood IgA nephropathy. J. Am. Soc. Nephrol..

[17] Kamei K., Nakanishi K., Ito S., Saito M., Sako M., Ishikura K., Hataya H., Honda M., Iijima K., Yoshikawa N. (2011). Japanese Pediatric IgA Nephropathy Treatment Study Group. Long-term results of a randomized controlled trial in childhood IgA nephropathy. Clin. J. Am. Soc. Nephrol..

[18] Foster B.J., Bernard C., Drummond K.N., Sharma A.K. (2000). Effective therapy for severe Henoch-Schonlein purpura nephritis with prednisone and azathioprine: A clinical and histopathologic study. J. Pediatr..

[19] Shin J.I., Park J.M., Shin Y.H., Kim J.H., Lee J.S., Kil Kim P., Jeong H.J. (2005). Can azathioprine and steroids alter the progression of severe Henoch-Schönlein nephritis in children?. Pediatr. Nephrol..

[20] Cambier A., Troyanov S., Tesar V., Coppo R., Validation Study of Oxford Classification (VALIGA) Group (2022). Indication for corticosteroids in IgA nephropathy: Validation in the European VALIGA cohort of a treatment score based on the Oxford classification. Nephrol. Dial. Transplant..

[21] Sarcina C., Tinelli C., Ferrario F., Pani A., De Silvestri A., Scaini P., Del Vecchio L., Alberghini E., Buzzi L., Baragetti I. (2016). Changes in Proteinuria and Side Effects of Corticosteroids Alone or in Combination with Azathioprine at Different Stages of IgA Nephropathy. Clin. J. Am. Soc. Nephrol..

